# Relationship of neighborhood and individual socioeconomic status on mortality among older adults: Evidence from cross-level interaction analyses

**DOI:** 10.1371/journal.pone.0267542

**Published:** 2022-05-19

**Authors:** Taehyun Kim

**Affiliations:** Department of Health Policy and Management, University of Maryland School of Public Health, College Park, Maryland, United States of America; Ruhr University Bochum, GERMANY

## Abstract

**Background:**

The influence of community context and individual socioeconomic status on health is widely recognized. However, the dynamics of how the relationship of neighborhood context on health varies by individual socioeconomic status is less well understood.

**Objective:**

To examine the relationship between neighborhood context and mortality among older adults and examine how the influence of neighborhood context on mortality differs by individual socioeconomic status, using two measures of income-level and homeownership.

**Research design and subjects:**

A retrospective study of 362,609 Medicare Advantage respondents to the 2014–2015 Medicare Health Outcomes Survey aged 65 and older.

**Measures:**

Neighborhood context was defined using the deciles of the Area Deprivation Index. Logistic regression was used to analyze mortality with interaction terms between income/homeownership and neighborhood deciles to examine cross-level relationships, controlling for age, gender, race/ethnicity, number of chronic conditions, obese/underweight, difficulties in activities of daily living, smoking status, and survey year. Predicted mortality rates by group were calculated from the logistic model results.

**Results:**

Low-income individuals (8.9%) and nonhomeowners (9.1%) had higher mortality rates compared to higher-income individuals (5.3%) and homeowners (5.3%), respectively, and the differences were significant across all neighborhoods even after adjustment. With regression adjustment, older adults residing in less disadvantaged neighborhoods showed lower predicted 2-year mortality among high-income (4.86% in the least disadvantaged neighborhood; 6.06% in the most disadvantaged neighborhood; difference p-value<0.001) or homeowning individuals (4.73% in the least disadvantaged neighborhood; 6.25% in the most disadvantaged neighborhood; difference p-value<0.001). However, this study did not observe a significant difference in predicted mortality rates among low-income individuals by neighborhood (8.7% in the least disadvantaged neighborhood; 8.61% in the most disadvantaged neighborhood; difference p-value = 0.825).

**Conclusions:**

Low-income or non-homeowning older adults had a higher risk of mortality regardless of neighborhood socioeconomic status. While living in a less disadvantaged neighborhood provided a protective association for higher-income or homeowning older adults, low-income older adults did not experience an observable benefit.

## Introduction

The relationship between neighborhood context and socioeconomic status (SES) on health is widely recognized [1,2]. Previous studies found that persons living in lower socioeconomic status (SES) neighborhoods have increased risks of worse health outcomes, including chronic care management and physical functioning [3–5], mental health [6–8], and mortality [9–13].

It is thought that neighborhoods impact health through both the physical and social environment [2]. The physical environment influences health through exposure to pollution, poor housing, public transportation, and local infrastructures such as food markets, recreational facilities, medical centers, and education [9]. Social environment influences health through social cohesion, collective efficacy, and the availability of services that promote social organizations or social interaction [1]. For older adults, the role of neighborhoods on health may be even more important because they have less mobility and smaller social networks, making one more dependent on the local neighborhood [1,3,4,6,8,14,15]. Among older adults, previous studies have found that living in lower SES neighborhoods is associated with greater depressive symptoms [6,8,16] and with poorer physical health [5,15].

It is important to note that health is also strongly correlated with individual socioeconomic status. Low income has been consistently linked with a higher risk of mortality [17–19] and shorter life expectancy [20]. The associations between low-income and poorer health persist among older adults [15,21,22]. As well as income, homeownership is also widely appreciated its association with health conditions, including better self-rated health [23,24], lower likelihood of depression [25], and lower risks of mortality [24,26]. The dynamics of neighborhood and individual-level SES are not well understood. It is thought that higher SES neighborhoods could ameliorate the adverse effect of low individual SES on health by providing a richer physical and social environment [27,28]. On the other hand, low SES individuals residing in high SES neighborhoods could experience higher risks to their health due to exacerbated relative poverty and lack of access to expensive neighborhood resources [10,11,15].

Upon the mixed evidence in the literature, this study examined the cross-level interaction of neighborhood and individual SES on mortality, in the context of Medicare Advantage (MA) beneficiaries responding to the Medicare Health Outcomes Survey (HOS) in 2014 and 2015. This study used two measures of individual-level SES: annual income and wealth (homeownership). Income is an essential measure of SES that is known to have a large influence on health [15,27,29], but income may not solely represent one’s economic conditions, particularly among older adults [25,30]. Income tends to generally decrease later in the life course, which may result in smaller variations in income inequality. In addition, income and wealth may not substitute each other interchangeably [25,26]. Also, homeownership is considered to reflect the lifetime income or accumulated past savings [24,26,30]. Thus, this study used homeownership as a measure for the wealth aspect of individual SES, as well as annual income.

The objective of this study was (1) to examine the association of neighborhood SES, represented by the Area Deprivation Index (ADI), and individual SES, defined by self-reported household income and homeownership, with mortality among older adults (2) to determine how the relationship of neighborhood SES may differ by individual SES. The findings of this study would contribute to the understanding of the cross-level dynamics of individual SES and area-level measures.

## Materials and methods

### Data

This study used the Medicare HOS baseline surveys fielded in 2014 and 2015. These were the most recent survey data years including 2-year mortality data for the survey sample at the time of research. The survey was administered to eligible MA beneficiaries via telephone and mail. The Centers for Medicare and Medicaid Services requires all MA contracts with more than 500 enrollees to field the HOS to inform quality improvement and performance assessment.

A total of 1,212,742 were eligible for the 2014 and 2015 HOS baseline survey. Among those eligible, a total of 587,887 responded to the baseline survey (response rate = 48.5%). This study excluded survey respondents younger than 65 (n = 98,699), individuals living in Puerto Rico, Virgin Islands, and Guam (n = 11,815), respondents in Alaska and Wyoming due to their small sample size (n = 80), those whose ADI values were not matched (n = 8,552). Out of 468,741 participants, 140,048 had missing values with income, and 71,219 had missing values with homeownership. Further excluding those who had any missing values with control variables (n = 26,127 for income sample, n = 34,913 for homeownership sample), the final analytic sample had 302,566 respondents for the income model, and 362,609 respondents for the homeownership model. Sample flow diagram is provided in S1 Fig.

### Measurement

The dependent variable was mortality. Mortality was measured by an administrative item from Performance Measurement data published after the baseline survey that indicates a beneficiary’s death within the 2-year window from the baseline survey [31].

The primary independent variables were measures of individual SES (income, homeownership) and neighborhood socioeconomic status. First, income was measured by a survey item asking, “*Which of the following categories best represents the combined income for all family members in your household for the past 12 months*?” “Low-income” status was defined as lower than $20,000 of household income, and “higher-income” status was defined as the rest of the respondents with $20,000 of household income or higher. After preliminary analyses on income and mortality, dichotomizing the income variable into low-income and higher-income was considered most appropriate for the objective of this analysis.

Second, homeownership was defined by the survey item asking the ownership of the house or apartment they were living in. Homeowners were identified as those who answered living in a house or apartment owned or being bought by themselves. Those who answered they were living in a place owned or bought by someone in the family other than themselves, rented for money, or not owned and living without payment of rent were identified as nonhomeowners.

Neighborhood-level SES was defined by the ADI, which is a composite measure that defines neighborhood socioeconomic status by the level of disadvantage at the Census block group level. HOS respondent’s 9-digit zip code was used to link to the Census block group. ADI includes 17 factors collected by the U.S. Census including poverty, education, employment, and housing quality [32,33]. High scores of ADI indicate the neighborhood is less disadvantaged, and low scores of ADI indicate more disadvantaged. Neighborhood status was defined using ADI deciles based on the US population: ADI decile group 1 (least disadvantaged group) to ADI decile group 10 (most disadvantaged group).

Other covariates were selected based on the Aday-Anderson health behavior model [34]. Predisposing factors included age (65–70, 70–75, 75–80, 80–85, 85+), sex (female, male), and self-reported race/ethnicity (non-Hispanic white, non-Hispanic Black, Hispanic, non-Hispanic Asian, other). Enabling factors were reflected in the primary independent variables as income and neighborhood status, so no other enabling factors were included. Need factors included physical/mental health status and risk factors: BMI, number of chronic conditions, difficulties in activities of daily living (ADL), and smoking status. BMI was categorized as obese (BMI >30) and underweight (BMI <20), following the survey’s categorization. A total of 14 self-reported chronic conditions (high blood pressure, angina pectoris/coronary artery disease, congestive heart failure, myocardial infarction/heart attack, other heart conditions, stroke, emphysema/asthma/chronic obstructive pulmonary disease, Crohn’s disease/ulcerative colitis/inflammatory bowel disease, arthritis hip/knee/hand/wrist, osteoporosis, sciatica, diabetes, depression, and cancer) were grouped into none, 1–2 conditions, 3–5 conditions, and 6 or more conditions, based on the preliminary analysis of the bivariate distribution between the number of chronic conditions and mortality, which suggested the relationship was not linear.

For functional limitations, respondents were identified as ‘having difficulties in 1 or more activities of daily living (ADLs) if they answered they have difficulty doing or are unable to do one or more of the following: bathing, dressing, eating, getting in or out of chairs, walking, and using the toilet. Smoking status was identified as 1 if the respondent answered every day or some days to the survey item asking whether they smoke every day, some days, or not at all. Lastly, the year dummy variable was included in the model to adjust the possible differences due to the survey year.

### Analysis

Descriptive statistics are provided to describe the sample characteristics stratified by income for Model 1 and homeownership for Model 2, and chi-2 tests were performed to examine whether higher-income and low-income individuals (homeowners and nonhomeowners) had different characteristics. Then, this study examined the relationship between individual SES (income, homeownership) and neighborhood status on mortality using logistic regressions with standard errors clustered at the state level to control the potential correlations between observations within states with interaction terms between income/homeownership and ADI deciles. To examine cross-level interactions between neighborhood and individual level SES, the predicted probabilities were calculated from a logistic model with the interaction term between income and ADI deciles and another model with the interaction term between homeownership and ADI deciles [35]. The predicted probabilities were compared between groups using chi-2 tests.

As a sensitivity analysis, multiple imputations were implemented to address missing values, and the results are shown in S1 Table (descriptive statistics) and S2 Table (regression results). The multiple imputation analysis used a chained equations method with logistic regressions for binary variables (income, homeownership, difficulties in ADL, smoking status) and multinomial logistic models for categorial variables (race/ethnicity, the number of chronic conditions, BMI). The variables of 2-year mortality, age, gender, survey year, and ADI were not imputed as they are administered variables rather than self-reported. The analysis was conducted using Stata IC ver. 16. This study was determined exempt from IRB review by University of Maryland IRB [1292614–2].

## Results

Table 1 shows the descriptive statistics of the sample and the p-values from the chi-2 tests. In the study sample, 6.6% died within two years of the baseline survey. Low-income respondents had a higher 2-year mortality rate than higher-income respondents (8.9% versus 5.3%). One-third (35.8%) reported household incomes less than $20,000; 5.8% of low-income respondents and 8.9% of higher-income respondents resided in the least disadvantaged neighborhood (ADI deciles group 1); 14.1% of low-income respondents and 3.8% of higher-income respondents resided in the most disadvantaged neighborhood (ADI deciles group 10).

**Table 1:** Descriptive statistics.

	Model 1: Income				Model 2: Homeownership			
	Overall	Higher-Income	Low-Income	p	Overall	Homeowner	Nonhomeowner	p
n (%)	302566	194124 (64.2)	108442 (35.8)		362609	247545 (68.3)	115064 (31.7)	
Died (%)	19847 (6.6)	10247 (5.3)	9600 (8.9)	<0.001	23516 (6.5)	13043 (5.3)	10473 (9.1)	<0.001
ADI decile (%)				<0.001				<0.001
ADI group 1 (least disadvantaged)	23513 (7.8)	17220 (8.9)	6293 (5.8)		28457 (7.8)	18722 (7.6)	9735 (8.5)	
ADI group 2	28681 (9.5)	22070 (11.4)	6611 (6.1)		34645 (9.6)	24381 (9.8)	10264 (8.9)	
ADI group 3	34726 (11.5)	26598 (13.7)	8128 (7.5)		41597 (11.5)	30375 (12.3)	11222 (9.8)	
ADI group 4	36342 (12.0)	26704 (13.8)	9638 (8.9)		43331 (11.9)	31812 (12.9)	11519 (10.0)	
ADI group 5	36876 (12.2)	25956 (13.4)	10920 (10.1)		43827 (12.1)	32050 (12.9)	11777 (10.2)	
ADI group 6	34629 (11.4)	22739 (11.7)	11890 (11.0)		41528 (11.5)	30072 (12.1)	11456 (10.0)	
ADI group 7	31775 (10.5)	18908 (9.7)	12867 (11.9)		37793 (10.4)	26156 (10.6)	11637 (10.1)	
ADI group 8	28422 (9.4)	15113 (7.8)	13309 (12.3)		33890 (9.3)	22693 (9.2)	11197 (9.7)	
ADI group 9	24999 (8.3)	11453 (5.9)	13546 (12.5)		30138 (8.3)	19126 (7.7)	11012 (9.6)	
ADI group 10 (most disadvantaged)	22603 (7.5)	7363 (3.8)	15240 (14.1)		27403 (7.6)	12158 (4.9)	15245 (13.2)	
Age (%)				<0.001				<0.001
65–69	96789 (32.0)	65150 (33.6)	31639 (29.2)		112622 (31.1)	77353 (31.2)	35269 (30.7)	
70–74	83967 (27.8)	56921 (29.3)	27046 (24.9)		100010 (27.6)	71143 (28.7)	28867 (25.1)	
75–79	56567 (18.7)	35402 (18.2)	21165 (19.5)		68868 (19.0)	48083 (19.4)	20785 (18.1)	
80–84	36618 (12.1)	21714 (11.2)	14904 (13.7)		45577 (12.6)	30718 (12.4)	14859 (12.9)	
85+	28625 (9.5)	14937 (7.7)	13688 (12.6)		35532 (9.8)	20248 (8.2)	15284 (13.3)	
Gender (%)				<0.001				<0.001
Female	170944 (56.5)	98548 (50.8)	72396 (66.8)		210872 (58.2)	136191 (55.0)	74681 (64.9)	
Race/Ethnicity (%)				<0.001				<0.001
White	226405 (74.8)	163327 (84.1)	63078 (58.2)		266418 (73.5)	201484 (81.4)	64934 (56.4)	
Black	27611 (9.1)	10548 (5.4)	17063 (15.7)		12989 (3.6)	5932 (2.4)	7057 (6.1)	
Hispanic	29026 (9.6)	10346 (5.3)	18680 (17.2)		35520 (9.8)	17006 (6.9)	18514 (16.1)	
Asian	10762 (3.6)	5515 (2.8)	5247 (4.8)		36644 (10.1)	16782 (6.8)	19862 (17.3)	
Other	8762 (2.9)	4388 (2.3)	4374 (4.0)		11038 (3.0)	6341 (2.6)	4697 (4.1)	
# of chronic conditions (%)				<0.001				<0.001
None	24815 (8.2)	18460 (9.5)	6355 (5.9)		30317 (8.4)	23235 (9.4)	7082 (6.2)	
1 to 2	112413 (37.2)	80066 (41.2)	32347 (29.8)		135801 (37.5)	100746 (40.7)	35055 (30.5)	
3 to 5	127770 (42.2)	78171 (40.3)	49599 (45.7)		152473 (42.0)	100420 (40.6)	52053 (45.2)	
6 or more	37568 (12.4)	17427 (9.0)	20141 (18.6)		44018 (12.1)	23144 (9.3)	20874 (18.1)	
BMI (%)				<0.001				<0.001
Normal/Overweight	194978 (64.4)	129560 (66.7)	65418 (60.3)		234016 (64.5)	164469 (66.4)	69547 (60.4)	
Obese	93599 (30.9)	57017 (29.4)	36582 (33.7)		111370 (30.7)	72684 (29.4)	38686 (33.6)	
Underweight	13989 (4.6)	7547 (3.9)	6442 (5.9)		17223 (4.7)	10392 (4.2)	6831 (5.9)	
Difficulties in ADL (%)				<0.001				<0.001
1+	105911 (35.0)	53165 (27.4)	52746 (48.6)		125144 (34.5)	69999 (28.3)	55145 (47.9)	
Smoking status (%)				<0.001				<0.001
Smoke	30612 (10.1)	15530 (8.0)	15082 (13.9)		35859 (9.9)	20440 (8.3)	15419 (13.4)	
Survey year (%)								<0.001
2015	143228 (47.3)	94776 (48.8)	48452 (44.7)	<0.001	171630 (47.3)	118333 (47.8)	53297 (46.3)	

Source: Medicare Health Outcomes Survey, 2014–2015.

As for homeownership, slightly less than a third (31.7%) were nonhomeowners, and they had a higher 2-year mortality rate (9.1%) than homeowners (5.3%). Similar proportions of respondents were living in the least advantaged neighborhood (ADI deciles group 1) among homeowners (7.6%) and nonhomeowners (8.5%), but the proportions of those living in the most disadvantaged neighborhood (ADI deciles group 10) differed by almost three times (homeowners 4.9% vs. nonhomeowners 13.2%).

The results indicate that lower-income and not homeowning respondents were significantly more likely to be older, female, racial/ethnic minority, either obese or under-weight, to have more chronic conditions and difficulties in ADLs, and smokers compared to higher-income and homeowning respondents.

Table 2 shows the logistic regression results on mortality rates including interaction terms between neighborhood status and individual SES (income, homeownership). The full results with covariates is reported in S5 Table. The first two columns show the model using income as the measure of individual SES (Model 1), and the last two columns show the model using homeownership as the measure of individual SES (Model 2).

**Table 2:** Logistic regression results on 2-year mortality.

	Model1: Income		Model 2: Homeownership	
Variables	Odds Ratio	95% Confidence Interval	Odds Ratio	95% Confidence Interval
Income level [ref: higher-income]				
Low-income	1.40***	(1.19–1.65)	-	-
Homeownership [ref: homeowner]				
Nonhomeowner	-	-	1.47***	(1.32–1.63)
ADI decile [ref: group 1 (least disadvantaged)]				
ADI group 2	0.95	(0.87–1.04)	0.93	(0.84–1.03)
ADI group 3	0.97	(0.87–1.07)	0.99	(0.89–1.10)
ADI group 4	1.05	(0.96–1.15)	1.08*	(0.99–1.19)
ADI group 5	1.14***	(1.04–1.25)	1.12**	(1.01–1.25)
ADI group 6	1.14***	(1.04–1.24)	1.15***	(1.04–1.28)
ADI group 7	1.13**	(1.03–1.25)	1.17***	(1.07–1.27)
ADI group 8	1.28***	(1.13–1.44)	1.28***	(1.14–1.43)
ADI group 9	1.29***	(1.17–1.41)	1.33***	(1.21–1.47)
ADI group 10 (most disadvantaged)	1.29***	(1.16–1.44)	1.38***	(1.20–1.58)
Interaction				
Lower indiv. SES * ADI group 2	1.06	(0.95–1.19)	1.09	(0.95–1.25)
Lower indiv. SES * ADI group 3	1.04	(0.88–1.24)	1.05	(0.93–1.18)
Lower indiv. SES * ADI group 4	1.04	(0.88–1.22)	1.02	(0.92–1.13)
Lower indiv. SES * ADI group 5	0.92	(0.77–1.11)	1.02	(0.90–1.14)
Lower indiv. SES * ADI group 6	0.89	(0.73–1.09)	0.96	(0.83–1.11)
Lower indiv. SES * ADI group 7	0.91	(0.76–1.09)	0.94	(0.85–1.05)
Lower indiv. SES * ADI group 8	0.82**	(0.69–0.98)	0.94	(0.82–1.07)
Lower indiv. SES * ADI group 9	0.75***	(0.62–0.92)	0.83***	(0.74–0.93)
Lower indiv. SES * ADI group 10	0.77***	(0.65–0.90)	0.76***	(0.62–0.92)
Observations	302,566		362,609	

Note:

*p<0.1

**p<0.05

***p<0.01.

Source: Medicare Health Outcomes Survey, 2014–2015. Note: The models were controlled for age (65–69, 70–74, 80–84, 85 or older), sex (male, female), race/ethnicity (non-Hispanic white, non-Hispanic Black, non-Hispanic Asian, Hispanic, other), the number of chronic conditions (none, 1–2, 3–5, 6 or more), BMI (normal/overweight, obese, underweight), difficulties in ADL (none, 1 or more), smoking status (not smoking, smoking), and survey year (2014, 2015).

Those who had low income (odds ratio [OR]: 1.40, 95% confidence interval [CI]: 1.19–1.65) or were nonhomeowners (OR: 1.47, 95% CI: 1.32–1.63) had higher odds of 2-year mortality than those with higher income or homeownership. Living in a higher ADI decile neighborhood was associated with higher risks of death in both models; for example, living in a neighborhood of ADI decile group 10 had an odds ratio of 1.29 (95% CI: 1.16–1.44) of 2-year mortality in Model 1 and 1.38 (95% CI: 1.20–1.58) in Model 2. The interaction terms between individual SES and neighborhood status had decreasing odds of mortality as it goes to higher ADI decile groups. For example, in both models, the interaction terms of ADI groups 2–7 were not significantly different from the interaction term of ADI group 1, and the interaction terms of ADI groups 8–10 are significant with odds ratios smaller than 1. The logistic regression results without the interaction terms are provided in S5 Table, side by side with the results of the models with the interaction terms. The two sets of results suggest that the full picture on the cross-level relationships between individual-level and neighborhood-level SES is not captured without the interaction terms in the model.

For other covariates, older age was associated with higher mortality, and the magnitude increased according to age in both models. Living with more chronic conditions contributed to a higher risk of mortality. ADL deficits and smoking were also associated with higher mortality rates. Protective factors include being female, non-Hispanic Black (only significant among the low-income), Hispanic, or Asian compared to non-Hispanic white, and obese (S5 Table).

**Fig 1:**
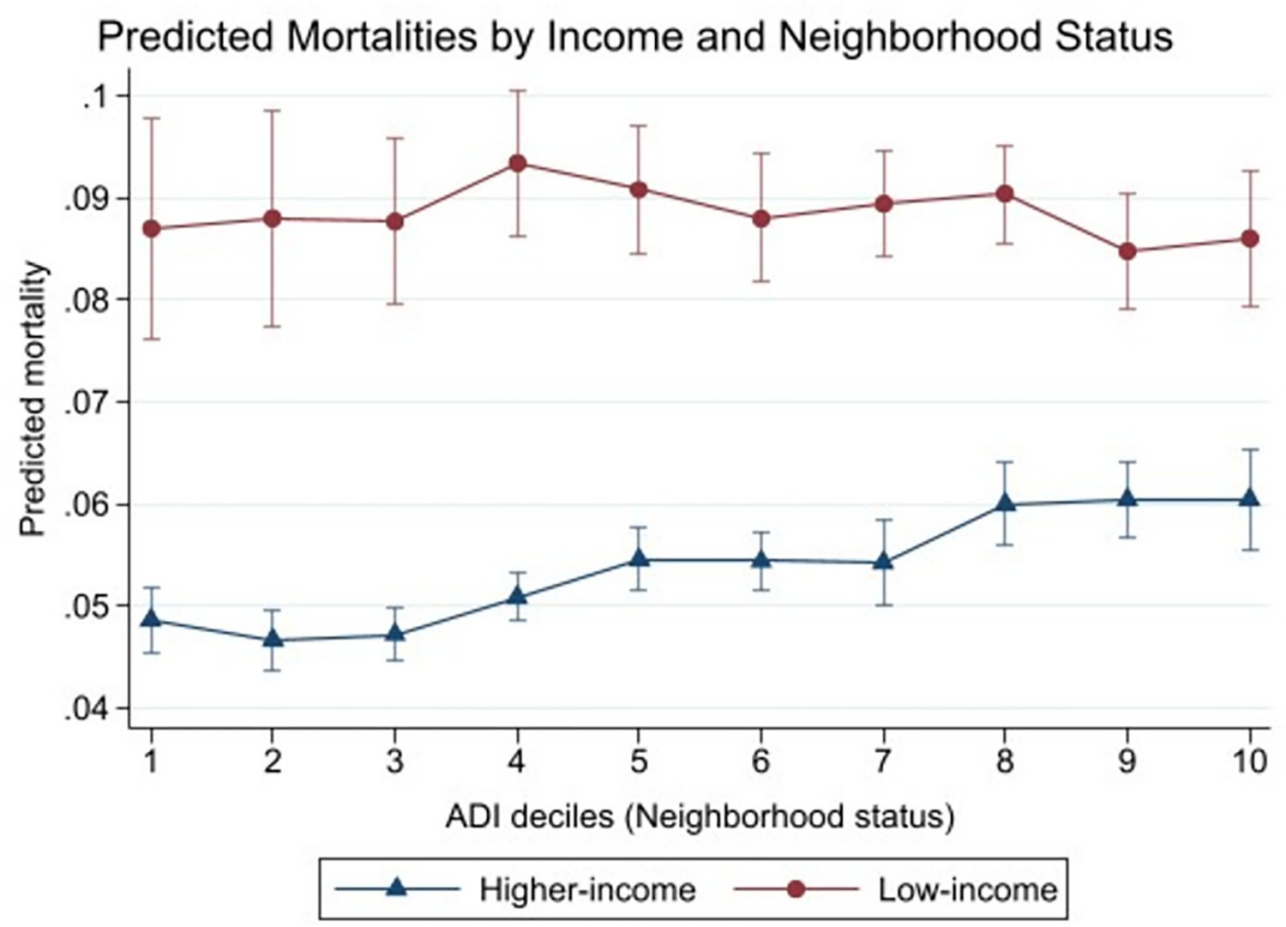
Predicted mortality rates by ADI decile groups, higher-income vs. low-income among older adults ages 65+. Source: Medicare Health Outcomes Survey, 2014–2015. Note: The predicted mortalities were obtained based on the regression models presented in Table 2. The models were controlled for age (65–69, 70–74, 80–84, 85 or older), sex (male, female), race/ethnicity (non-Hispanic white, non-Hispanic Black, non-Hispanic Asian, Hispanic, other), the number of chronic conditions (none, 1–2, 3–5, 6 or more), BMI (normal/overweight, obese, underweight), difficulties in ADL (none, 1 or more), smoking status (not smoking, smoking), and survey year (2014, 2015).

**Fig 2:**
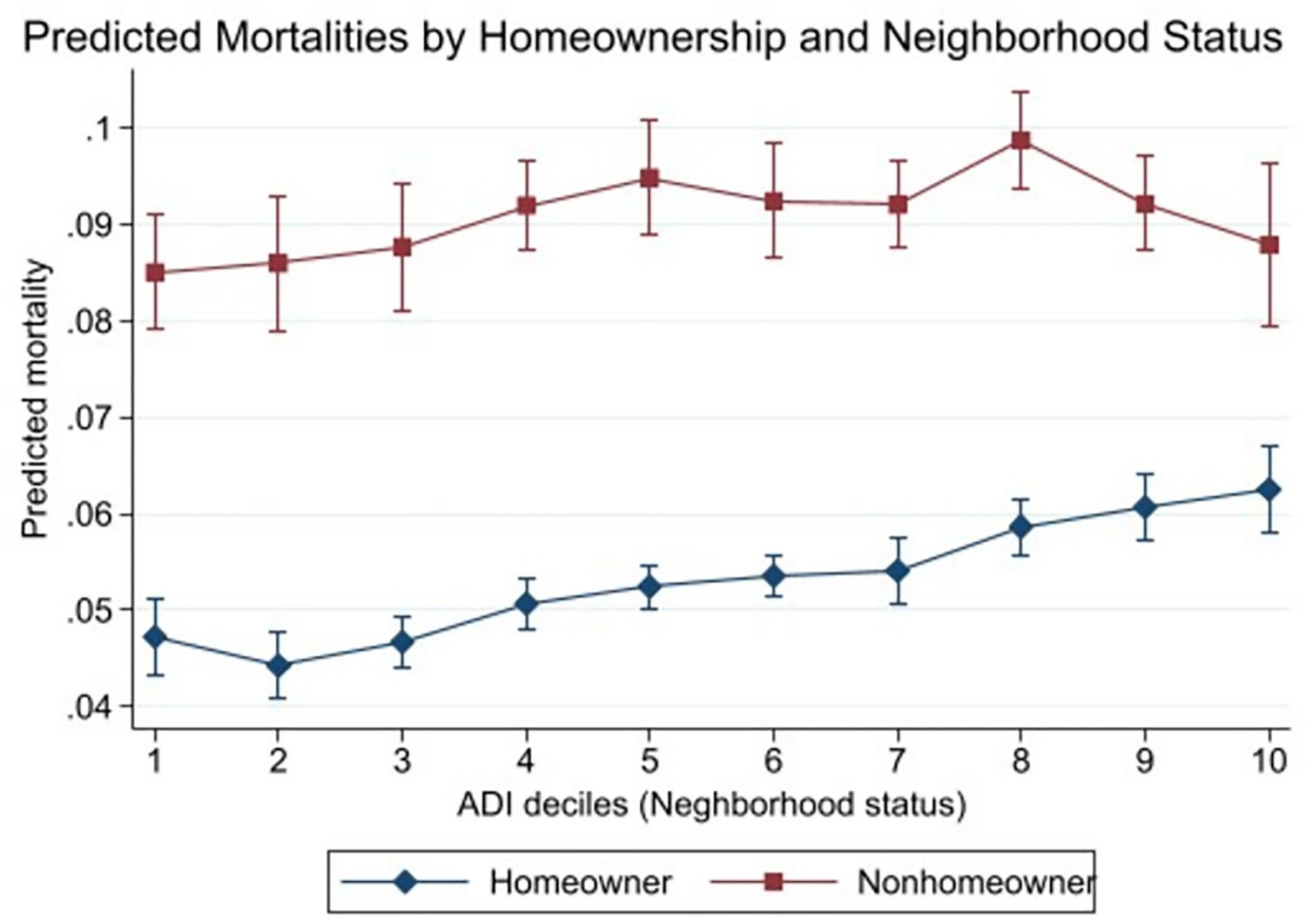
Predicted mortality rates by ADI decile groups, homeowners vs. nonhomeowners among older adults ages 65+. Source: Medicare Health Outcomes Survey, 2014–2015. Note: The predicted mortalities were obtained based on the regression models presented in Table 2. The models were controlled for age (65–69, 70–74, 80–84, 85 or older), sex (male, female), race/ethnicity (non-Hispanic white, non-Hispanic Black, non-Hispanic Asian, Hispanic, other), the number of chronic conditions (none, 1–2, 3–5, 6 or more), BMI (normal/overweight, obese, underweight), difficulties in ADL (none, 1 or more), smoking status (not smoking, smoking), and survey year (2014, 2015).

To further examine the cross-level interaction between individual SES and neighborhood status, the adjusted predicted mortalities are displayed in Table 3 and Figs 1 and 2. Table 3 Panel A and Fig 1 show the predicted mortality rates among higher-income and low-income by ADI decile groups, and Table 3 and Fig 2 illustrate among homeowners and nonhomeowners. In Table 3, the first four columns show the higher individual SES group’s the predicted mortality rates and comparisons between ADI group 1 and higher ADI groups within the same individual SES group (for example, comparing higher-income ADI group 1 vs. higher-income ADI group 7). The next four columns shoe the lower individual SES group’s information. The two right-most columns display the differences and the p-values comparing the predicted mortalities between higher SES and lower SES groups within the same ADI group (for example, comparing homeowners in ADI group 3 vs. nonhomeowners in ADI group 3).

**Table 3:** Predicted mortality rates by individual and neighborhood SES.

**Panel A: Income**										
	**Higher-Income**				**Low-Income**				**Difference Higher-Income vs. Low-Income**	
	Predicted mortality (%)	Std. error	**Comparison** **vs. ADI group 1**		Predicted mortality (%)	Std. error	**Comparison** **vs. ADI group 1**		Difference (%point)	P-value
			Difference (%point)	P-value			Difference (%point)	P-value		
ADI group 1	4.86	0.16	-	-	8.7	0.55	-	-	3.84	<0.001
ADI group 2	4.67	0.15	-0.19	0.305	8.8	0.54	0.10	0.732	4.13	<0.001
ADI group 3	4.72	0.13	0.14	0.537	8.76	0.42	0.06	0.894	4.04	<0.001
ADI group 4	5.09	0.12	0.20	0.257	9.35	0.36	0.65	0.192	4.26	<0.001
ADI group 5	5.45***	0.16	0.59	0.006	9.08	0.32	0.38	0.498	3.63	<0.001
ADI group 6	5.44***	0.14	0.58	0.003	8.8	0.32	0.10	0.886	3.36	<0.001
ADI group 7	5.42**	0.22	0.56	0.014	8.96	0.26	0.26	0.671	3.54	<0.001
ADI group 8	6.00***	0.21	1.14	<0.001	9.04	0.24	0.34	0.567	3.04	<0.001
ADI group 9	6.05***	0.19	1.19	<0.001	8.48	0.29	0.22	0.712	2.43	<0.001
ADI group 10	6.06***	0.25	1.20	<0.001	8.61	0.34	-0.09	0.825	2.55	<0.001
**Panel B: Homeownership**										
	**Homeowner**				**Nonhomeowner**				**Difference Homeowner vs. Nonhomeowner**	
	Predicted mortality (%)	Std. error	**Comparison vs. ADI group 1**		Predicted mortality (%)	Std. error	**Comparison vs. ADI group 1**		Difference (%point)	P-value
			Difference (%point)	P-value			Difference (%point)	P-value		
ADI group 1	4.73	0.21	-	-	8.51	0.31	-	-	3.78	<0.001
ADI group 2	4.42	0.17	-0.31	0.15	8.6	0.36	0.09	0.74	4.18	<0.001
ADI group 3	4.67	0.13	-0.06	0.82	8.77	0.33	0.26	0.36	4.10	<0.001
ADI group 4	5.07*	0.14	0.34	0.08	9.2**	0.24	0.61	0.012	4.13	<0.001
ADI group 5	5.24**	0.11	0.51	0.02	9.49***	0.3	0.98	0.002	4.25	<0.001
ADI group 6	5.36***	0.1	0.63	0.005	9.24*	0.3	0.73	0.06	3.88	<0.001
ADI group 7	5.41***	0.18	0.68	<0.001	9.21**	0.23	0.70	0.04	3.80	<0.001
ADI group 8	5.87***	0.15	1.14	<0.001	9.87***	0.26	1.36	0.002	4.00	<0.001
ADI group 9	6.07***	0.18	1.34	<0.001	9.21**	0.25	0.70	0.03	3.14	<0.001
ADI group 10	6.25***	0.23	1.52	<0.001	8.79	0.43	0.28	0.44	2.54	<0.001

Note:

*** p<0.01

** p<0.05

* p<0.1. The p-values are from chi-2 tests comparing the differences between groups. The predicted mortalities were obtained based on the regression models presented in Table 2. The models were controlled for age (65–69, 70–74, 80–84, 85 or older), sex (male, female), race/ethnicity (non-Hispanic white, non-Hispanic Black, non-Hispanic Asian, Hispanic, other), the number of chronic conditions (none, 1–2, 3–5, 6 or more), BMI (normal/overweight, obese, underweight), difficulties in ADL (none, 1 or more), smoking status (not smoking, smoking), and survey year (2014, 2015). Source: Medicare Health Outcomes Survey, 2014–2015.

### Income

#### Differences within higher-income respondents

Predicted mortalities increased as ADI deciles increase, i.e., as respondents lived in more disadvantaged neighborhoods (Table 3 Panel A columns 2–4). While the predicted mortality rates of higher-income individuals in the least disadvantaged neighborhood was 4.86% (standard error [se]: 0.16), those in the most disadvantaged neighborhood had a significantly higher risk of death (6.06%, se: 0.25). The predicted mortalities in ADI groups 2, 3, and 4 were not significantly different from the predicted mortality of the ADI group 1, but higher ADI groups, indicating greater neighborhood disadvantage, had significantly higher predicted mortality than ADI group 1. In Fig 1, the plotted line of the higher-income is increasing as ADI deciles increase, with two nearly flat sections in ADI deciles 5–7 and ADI deciles 8–10.

#### Differences within low-income respondents

Unlike among higher-income respondents, the trend of predicted mortalities among the low-income was not increasing with ADI deciles (Table 3 Panel A columns 5–7). The predicted mortalities remain rather constant across all the ADI groups ranging from 8.70% in ADI group 1 (least disadvantaged neighborhood) to 8.61% in ADI group 10 (most disadvantaged neighborhood). In all ADI groups 2–10, the predicted mortality was not significantly different from the predicted mortality in ADI group 1.

#### Differences between the higher-income and the low-income

Across all ADI groups, the predicted mortality rates of low-income individuals were significantly higher than those of higher-income individuals (Table 3 Panel A column 8). The differences between higher-income and low-income individuals in the same ADI group were greater than both the differences by ADI groups within the higher-income and within the low-income.

### Homeownership

#### Differences within homeowner respondents

Similar to the results using income measure, when using homeownership measure, the predicted mortalities of the higher individual SES group increased as respondents lived in more disadvantaged neighborhoods (i.e., higher ADI decile groups) (Table 3 Panel B columns 2–4, Fig 2). Among homeowners, those who lived in ADI group 1 had a mortality rate of 4.73% (se: 0.21), which slightly decreased among those who lived in ADI group 2 and kept increasing for higher ADI decile groups to 6.25% in ADI group 10 (se: 0.23). Compared to the ADI group 1, the predicted mortalities were significantly higher among ADI groups 5–10.

#### Differences within nonhomeowner respondents

Again, the changes in predicted mortality by ADI among the lower individual-SES group did not show the same pattern of changes by ADI among the higher individual-SES group when using homeownership measure (Table 3 Panel B columns 4–7). However, it was also different from the trend of mortality rates among the low-income group; that is, nonhomeowners’ mortality rates neither increased as they lived in more disadvantaged neighborhoods (higher ADI group) nor remained constant. Rather, the predicted mortality among nonhomeowners increased from ADI group 1 (8.51%) to ADI group 5 (9.49%), then it slightly decreased for ADI groups 6 (9.24%) and 7 (9.21%), had a spike for ADI group 8 (9.87%), which then decreased again in ADI group 9 (9.21%) and ADI group 10 (8.79%).

#### Differences between the homeowners and the nonhomeowners

Analogous to the model using income measure, the predicted mortalities of nonhomeowners were significantly higher than those of homeowners across all ADI groups (Table 3 Panel B column 8). The differences between homeowners and nonhomeowners in the same ADI group were greater than both the differences by ADI groups within homeowners and within nonhomeowners.

### Supplementary analysis

The descriptive statistics and regression results with multiple imputation are shown in S1 and S2 Tables. The multiple imputations were conducted on the main individual SES measure (income or homeownership) and covariates. Sample sizes after multiple imputations were 468,741 for both models. The results with multiple imputations did not have significant differences from the main analysis. The multiple imputation analysis suggests little evidence that missingness had significant impact on this paper’s findings.

However, the missing patterns presented in S3 and S4 Tables suggest a caveat with these results. Those with missing income values were more likely to have missing values with other variables as well, have died after 2 years of the baseline survey, and be older, compared to those with valid income values. This was also the case with those missing homeownership values when comparing with those with valid homeownership inputs. The ADI distributions were generally similar in both cases (income and homeownership).

## Discussion

In this study, this paper analyzed the cross-level effects of neighborhood SES and individual SES, using two measures (income and homeownership), on mortality with a large-sized, diverse sample of Medicare Advantage beneficiaries.

In the findings, first, neighborhood-level SES and individual-level SES were significantly associated with mortality rate. Mortality rates were significantly higher for low-income individuals and nonhomeowners than higher-income or homeowning individuals across neighborhoods in all ADI deciles. Second, the association of neighborhood context on mortality was significantly different by individual-SES (income and homeownership). Living in a less disadvantaged neighborhood did not attenuate the adverse effect of having lower individual SES, while among higher-income or homeowning individuals, living in less disadvantaged neighborhoods had consistent associations with lower risks of death compared to living in more disadvantaged neighborhoods. Third, the patterns of cross-level interactions of neighborhood context with individual SES were different when using income or homeownership as the measure of individual SES, especially among lower-SES.

The present paper’s findings are consistent with previous studies examining cross-level interactions between individual SES and neighborhood SES, when it comes to using income as the measure of individual SES. In a study of adults aged 25–74 years in California, Winkleby et al. (2006) found that the mortality of low-income individuals did not decrease when living in higher SES areas compared to when living in lower SES areas, unlike high-income individuals. Roos et al. (2004) also found different trends in mortality between low-income and high-income individuals aged 18–75 in Canada in disadvantaged and advantaged neighborhoods classified using various indicators such as neighborhood household income and education, and their results also showed that the differences in mortality rates based on income were greater than that the differences based on neighborhood status. This paper adds to the previous literature by focusing on a national sample of older adults enrolled in the Medicare Advantage program, and leveraging the large sample size to examine the relationship between mortality and individual/neighborhood SES at more granular levels.

One explanation for these results is that income is a stronger predictor of mortality than neighborhood SES among low-income older adults. Even though less disadvantaged neighborhoods may have more resources such as access to healthy foods, medical centers, and transportation, these resources may not be meaningful for low-income individuals if they cannot afford them. In addition, low-income individuals living in advantaged neighborhoods may experience higher relative disadvantage connected to social stigma overlaid on the low material level and a greater sense of deprivation [36].

As for homeownership, little is known about the cross-level interactions on health of homeownership with neighborhood SES. Previous studies have examined the relationships between homeownership and perception on neighborhood [37,38] or the relationships between homeownership and health outcomes [25,26,39,40], but not how homeownership interacts with neighborhood-level SES when affects health. This study contributes to the literature by examining the cross-level interactions between neighborhood SES and individual SES by using the measure of homeownership.

For the different patterns of associations among neighborhood SES, individual SES, and mortality between using income and using homeownership, Ross & Mirowsky (2008) argued that homeownership distinguishes economic well-being at the middle to high end of the continuum rather than the low. The nature of income and homeownership might operate differently with health among older adults, and further study is needed.

As for the decreasing mortality rates in ADI groups 9 and 10 among nonhomeowners, residents in more disadvantaged neighborhoods might have different characteristics than others. For example, residents in ADI groups 9 and 10 tend to be younger than residents in less disadvantaged neighborhoods. Residents in ADI group 10 were significantly more likely not to own the house they lived in (56.4%), compared to the other ADI groups (the highest proportion of nonhomeowners was 37.2%). These characteristics may have affected the decreased risks of mortality in ADI groups 9 and 10 among nonhomeowners.

Also, previous studies found that sense of control, residential stability, and perceived relative standing in the neighborhood mediates homeownership’s relationships with mental health and perceived neighborhood disorder [37–39]. These factors may operate differently for nonhomeowners in neighborhood SES decile groups.

The significant gaps between higher-SES and lower-SES individuals in predicted mortality rates indicate the importance of considering the cumulative effect of poverty. As incomes in older ages heavily rely on Social Security, especially among low-income older adults, and the Social Security income in one’s older ages reflect incomes in their younger ages [41,42], those who are low-income in older years are more likely to have been low-income during their younger years. Homeownership is also considered as the accumulation of lifetime economic living standards [26,30]. The accumulated financial hardship may have a much stronger impact on mortality in the sample than neighborhood disadvantage [43–45]. This paper’s results suggest the cumulative effects of poverty in the sample. Low-income older adults were twice as likely to be living with 6 or more chronic conditions (18.6% vs. 9%), more likely to be obese (33.7% vs. 29.4%), and more likely to be a current smoker (13.9% vs. 8%) than higher-income older adults.

The findings of this paper stress the significance of individual SES on health, which suggests the importance of reducing poverty and lifetime economic hardship in promoting the health of older adults. Policies that affect poverty often require efforts outside the health care sector, including but not limited to housing, transportation, education, environment, criminal justice [46]. Such cross-sectional collaborative efforts are needed at the federal, regional, state, and local levels. One successful example of cross-sectional effort at the local level is the Maryland Health Enterprise Zone (HEZ) initiative [47]. The HEZ initiative promoted collaborations between health departments, local government agencies, and local health care providers. They recruited primary care physicians to most underserved areas in the state and provided state-level tax incentives. The initiative also offered increased access to licensed clinical social workers to address the underserved population’s non-medical health needs [47,48]. In addition, programs to reduce poverty among older adults, such as programs to reduce copayments and out-of-pocket costs, may be effective to provide health benefits for lower-income individuals.

This study has several limitations. First, this study examined the relationship between neighborhood and individual SES in a sample of older adults Medicare Advantage beneficiaries. These results may not be generalized to older adults in the Medicare Fee-For-Service (FFS). Previous studies on differences between MA and FFS Medicare enrollees found that there were some but not substantial differences in sociodemographic characteristics (e.g., more racial minority, more low-income, more living in metropolitan area) [49,50], lower mortality rates among MA beneficiaries than FFS but decreasing over time enrolled [51], and fewer health care utilizations [49,50]. Despite some differences in population characteristics from FFS Medicare, Medicare Advantage plans account for one-third of all Medicare beneficiaries enrolling more than 20 million people, so it is important to examine these relationships with the MA beneficiaries’ program.

Second, this study is not able to provide which aspect of neighborhood disadvantage affects mortality and interacts with income level since this study uses a composite measure of neighborhood context. However, the composite index also helps us to capture the complex contributions of neighborhood resources and structure on mortality. Third, this study utilized self-reported survey measures for income, homeownership, and health status, which are subject to recall bias and respondent bias. Forth, the homeownership measure for wealth only captures whether the respondent was living in a house that they owned but not the value of the house if they owned it. There is also a possibility that someone did own a house but was not living in that place where they do not own, which is captured as non-homeowner as per how the survey asked homeownership. However, a study found that housing tenure (ownership or rent) had similar results to when using net wealth measure [52].

Though not without limitations, this paper has several strengths and contributions. This study adds to the literature by examining the cross-level interactions between neighborhood and individual SES, which has been less studied, using two measures of individual SES—income and homeownership. This study was able to apply the research question to a large-sized, diverse sample of older adults.

## Conclusion

Neighborhood-level SES and individual-level SES (income and homeownership) are significant predictors of mortality among older adults enrolled in MA plans. Income and homeownership were consistently a significant predictor of higher mortality across all neighborhood status. Among higher-income or homeowning individuals, mortality was higher in low SES neighborhoods compared to higher SES neighborhoods. On the other hand, among low-income or non-homeowning individuals, mortality did not increase with more disadvantaged neighborhood status. These findings suggest that the impact of neighborhood resources varies by individual income and homeownership, and approaches to improving health should recognize the differential impacts.

## Supporting information

S1 Fig Sample flow. Note: HOS: Health Outcomes Survey, ADI: Area Deprivation Index. Source: Medicare Health Outcomes Survey 2014–2015.
(JPG)

S1 Table Multiple imputed sample characteristics. Source: Medicare Health Outcomes Survey 2014–2015.
(DOCX)

S2 Table Regression results with multiple imputation. Note: *p<0.1; **p<0.05; ***p<0.01. Source: Medicare Health Outcomes Survey 2014–2015.
(DOCX)

S3 Table Descriptive characteristics comparing those with valid income values and those with missing income. Source: Medicare Health Outcomes Survey 2014–2015.
(DOCX)

S4 Table Descriptive characteristics comparing those with valid homeownership values and those with missing homeownership. Source: Medicare Health Outcomes Survey 2014–2015.
(DOCX)

S5 Table Regression results with and without interaction terms between income/homeownership and neighborhood status. Source: Medicare Health Outcomes Survey 2014–2015.
(DOCX)

S6 Table Bivariate analysis on income and mortality. Source: Medicare Health Outcomes Survey 2014–2015.
(DOCX)
