# Supplementary figures and images for: Relationship of neighborhood and individual socioeconomic status on mortality among older adults: Evidence from cross-level interaction analyses
Source: PLoS One. 2022 May 19;17(5):e0267542. doi: 10.1371/journal.pone.0267542 (PMC9119539; doi:10.1371/journal.pone.0267542)

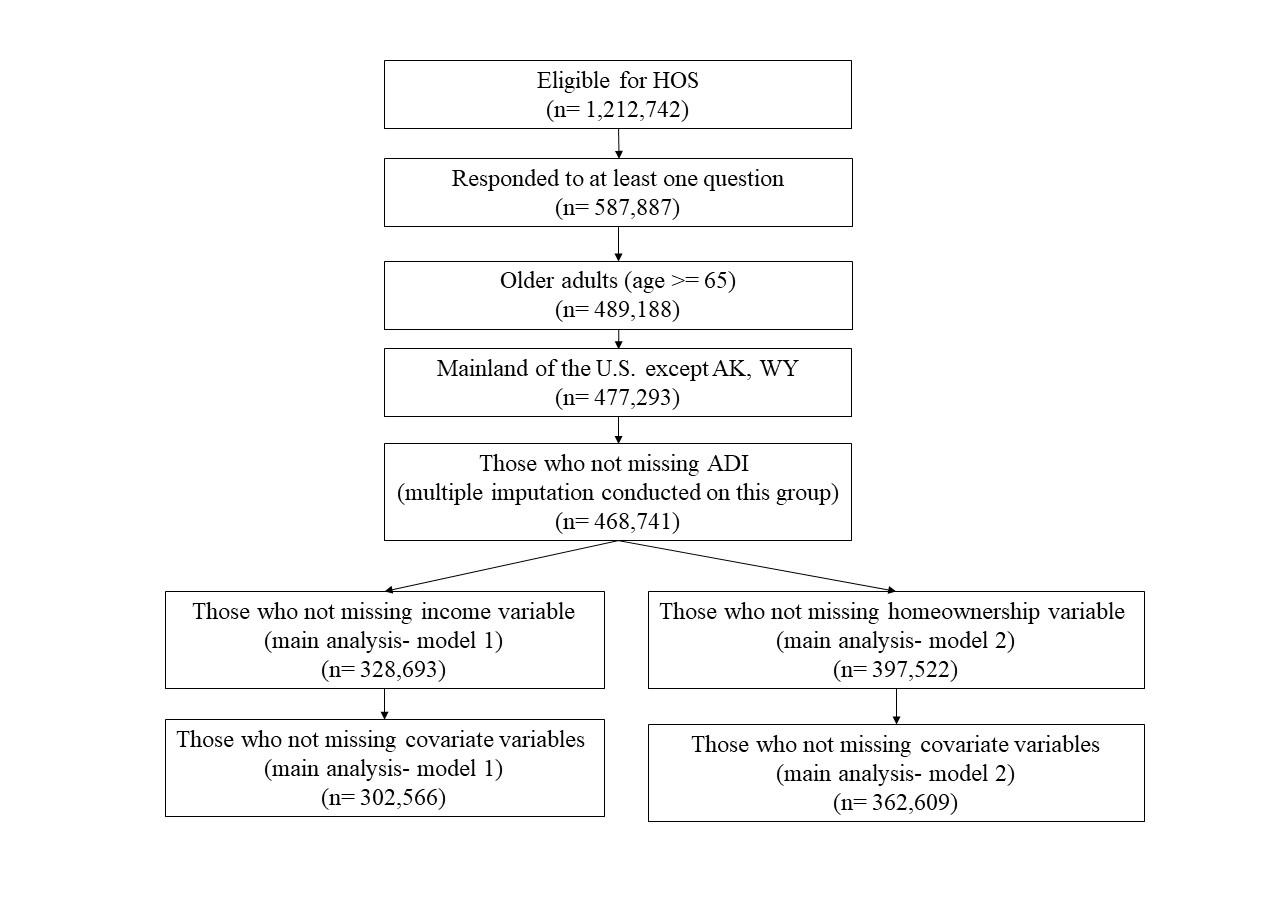

Supplement: S1 Fig — Note: HOS: Health Outcomes Survey, ADI: Area Deprivation Index. Source: Medicare Health Outcomes Survey 2014–2015. (JPG) [file pone.0267542.s001.jpg]
