# Supplementary material for: Relationship of neighborhood and individual socioeconomic status on mortality among older adults: Evidence from cross-level interaction analyses
Source: PLoS One. 2022 May 19;17(5):e0267542. doi: 10.1371/journal.pone.0267542 (PMC9119539; doi:10.1371/journal.pone.0267542)
Supplement: S1 Table — Source: Medicare Health Outcomes Survey 2014–2015. (DOCX) [file pone.0267542.s002.docx]

**S1 Table. Multiple imputed sample characteristics**

|  | Multiple Imputation (Income) | | |  | Multiple Imputation (Homeownership) | | |  |
| --- | --- | --- | --- | --- | --- | --- | --- | --- |
|  | Overall | Higher-income | Low-income | p | Overall | Homeowner | Nonhomeowner | p |
| n | 468741 | 290769 (62.0) | 177972 (38.0) |  | 468741 | 312303 | 156438 |  |
| Died (%) | 35650 (7.61) | 17867 (6.14) | 17783 (10.0) | <0.001 | 35650 (7.6) | 19035 (6.1) | 16615 (10.6) | <0.001 |
| ADI decile (%) |  |  |  | <0.001 |  |  |  | <0.001 |
| ADI group 1 | 37142 (7.9) | 26300 (9.0) | 10842 (6.1) |  | 37142 (7.9) | 23735 (7.6) | 13407 (8.6) |  |
| ADI group 2 | 44559 (9.5) | 33141 (11.4) | 11418(6.4) |  | 44559 (9.5) | 30403 (9.7) | 14156 (9.0) |  |
| ADI group 3 | 53244 (11.4) | 39550 (13.6) | 13694 (7.7) |  | 53244 (11.4) | 37982 (12.2) | 15262 (9.8) |  |
| ADI group 4 | 55154 (11.8) | 39621 (13.6) | 15533 (8.7) |  | 55154 (11.8) | 39661 (12.7) | 15493 (9.9) |  |
| ADI group 5 | 55824 (11.9) | 38425 (13.2) | 17399 (9.8) |  | 55824 (11.9) | 40071 (12.8) | 15753 (10.1) |  |
| ADI group 6 | 53106 (10.4) | 33996 (11.7) | 19110 (10.7) |  | 53106 (11.3) | 37734 (12.1) | 15372 (9.8) |  |
| ADI group 7 | 48840 (10.4) | 28229 (9.7) | 20611 (11.6) |  | 48840 (10.4) | 33213 (10.6) | 15627 (10.0) |  |
| ADI group 8 | 43856 (9.4) | 22596 (7.8) | 21260 (12.0) |  | 43856 (9.4) | 28870 (9.2) | 14986 (9.6) |  |
| ADI group 9 | 39345 (8.4) | 17348 (6.0) | 21997 (12.4) |  | 39345 (8.4) | 24471 (7.8) | 14874 (9.5) |  |
| ADI group 10 | 37671 (8.0) | 11563 (4.0) | 26108 (14.7) |  | 37671 (8.0) | 16163 (5.2) | 21508 (13.7) |  |
| Age (%) |  |  |  | <0.001 |  |  |  | <0.001 |
| 65-69 | 139224 (29.7) | 91439 (31.5) | 47785 (26.9) |  | 139224 (29.7) | 93798 (30.0) | 45426 (29.0) |  |
| 70-74 | 125694 (26.8) | 82906 (28.5) | 42788 (24.0) |  | 125694 (26.8) | 87655 (28.1) | 38039 (24.3) |  |
| 75-79 | 89152 (19.0) | 54204 (18.6) | 34948 (19.6) |  | 89152 (19.0) | 60963 (19.5) | 28189 (18.0) |  |
| 80-84 | 61424 (13.1) | 35292 (12.1) | 26132 (14.7) |  | 61424 (13.1) | 40416 (12.9) | 21008 (13.4) |  |
| 85+ | 53247 (11.4) | 26928 (9.3) | 26319 (14.8) |  | 53247 (11.4) | 29471 (9.4) | 23776 (15.2) |  |
| Female (%) | 275528 (58.8) | 153696 (52.9) | 121832 (68.5) | <0.001 | 275528 (58.8) | 173369 (55.5) | 102159 (65.3) | <0.001 |
| Race/Ethnicity (%) |  |  |  | <0.001 |  |  |  | <0.001 |
| White | 338070 (72.1) | 239958 (82.5) | 98112 (55.1) |  | 336580 (71.8) | 250952 (80.4) | 85628 (54.7) |  |
| Black | 47861 (10.2) | 17875 (6.2) | 29986 (16.9) |  | 18120 (3.9) | 7928 (2.5) | 10192 (6.5) |  |
| Hispanic | 50792 (10.8) | 17277 (5.9) | 33515 (18.8) |  | 48038 (10.2) | 22494 (7.2) | 25544 (16.3) |  |
| Asian | 17476 (3.7) | 8464 (2.9) | 9012 (5.1) |  | 51384 (11.0) | 22646 (7.3) | 28738 (18.4) |  |
| Other | 14542 (3.1) | 7195 (2.5) | 7347 (4.13) |  | 14619 (3.1) | 8283 (2.7) | 6336 (4.1) |  |
| # of chronic conditions (%) |  |  |  | <0.001 |  |  |  | <0.001 |
| None | 39472 (8.4) | 28367 (9.8) | 11105 (6.2) |  | 39523 (8.4) | 29636 (9.5) | 9887 (6.3) |  |
| 1 to 2 | 173717 (37.1) | 120140 (41.3) | 53577 (30.1) |  | 173324 (37.0) | 126055 (40.4) | 47269 (30.2) |  |
| 3 to 5 | 196781 (42.0) | 116245 (40.0) | 80536 (45.3) |  | 197014 (42.0) | 126577 (40.5) | 70437 (45.0) |  |
| 6 or more | 58771 (12.5) | 26017 (9.0) | 32754 (18.4) |  | 58880 (12.6) | 30035 (9.6) | 28845 (18.4) |  |
| BMI (%) |  |  |  | <0.001 |  |  |  | <0.001 |
| Normal/Overweight | 302430 (64.5) | 194406 (66.9) | 108024 (60.7) |  | 303389 (64.7) | 208082 (66.6) | 95307 (60.9) |  |
| Obese | 142904 (30.5) | 84250 (29.0) | 58654 (33.0) |  | 142680 (30.4) | 90978 (29.1) | 51702 (33.0) |  |
| Underweight | 23407 (5.0) | 12113 (4.2) | 11294 (6.4) |  | 22672 (4.8) | 13243 (4.2) | 9429 (6.0) |  |
| Difficulties in ADL (%) |  |  |  | <0.001 |  |  |  |  |
| 1+ | 169766 (36.2) | 81727 (28.1) | 88039 (49.5) |  | 169679 (36.2) | 92224 (29.5) | 77455 (49.5) | <0.001 |
| Smoking status (%) |  |  |  | <0.001 |  |  |  |  |
| Smoke | 45573 (9.7) | 22281 (7.7) | 23292 (13.1) |  | 45634 (9.7) | 25329 (8.1) | 20305 (13.0) | <0.001 |
| Survey year 2015 (%) | 228547 (48.8) | 146085 (50.2) | 82462 (46.33) | <0.001 | 228547 (48.8) | 153544 (49.2) | 75003 (47.9) | <0.001 |

Source: Medicare Health Outcomes Survey, 2014-2015. The p-values are from chi-2 tests comparing the groups (higher-income vs low-income, and homeowner vs. nonhomeowner).
