# Supplementary material for: Relationship of neighborhood and individual socioeconomic status on mortality among older adults: Evidence from cross-level interaction analyses
Source: PLoS One. 2022 May 19;17(5):e0267542. doi: 10.1371/journal.pone.0267542 (PMC9119539; doi:10.1371/journal.pone.0267542)
Supplement: S2 Table — Note: *p<0.1; **p<0.05; ***p<0.01. Source: Medicare Health Outcomes Survey 2014–2015. (DOCX) [file pone.0267542.s003.docx]

**S2 Table. Regression results with multiple imputation.**

|  | Model1: Income | | Model 2: Homeownership | |
| --- | --- | --- | --- | --- |
| Variables | Odds Ratio | 95% Confidence Interval | Odds Ratio | 95% Confidence Interval |
| Income level [ref: higher-income] |  |  |  |  |
| Low-income | 1.32*** | (1.17, 1.49) | - | - |
| Homeownership [ref: homeowner] |  |  |  |  |
| Nonhomeowner | - | - | 1.49 *** | (1.35, 1.64) |
| ADI decile [ref: group 1 (least disadvantaged)] |  |  |  |  |
| ADI group 2 | 0.93* | (0.86, 1.00) | 0.92 * | (0.84, 1.00) |
| ADI group 3 | 0.96 | (0.89, 1.04) | 0.98 | (0.90, 1.06) |
| ADI group 4 | 1.00 | (0.93, 1.07) | 1.05 | (0.97, 1.14) |
| ADI group 5 | 1.06 | (0.98, 1.13) | 1.08 * | (0.99, 1.16) |
| ADI group 6 | 1.06* | (0.99, 1.14) | 1.10 ** | (1.02, 1.18) |
| ADI group 7 | 1.03 | (0.96, 1.11) | 1.10 ** | (1.00, 1.20) |
| ADI group 8 | 1.15*** | (1.05, 1.26) | 1.18 *** | (1.09, 1.27) |
| ADI group 9 | 1.14*** | (1.06, 1.24) | 1.20 *** | (1.10, 1.30) |
| ADI group 10 (most disadvantaged) | 1.20*** | (1.08, 1.33) | 1.27 *** | (1.15, 1.40) |
| Interaction |  |  |  |  |
| Lower indiv. SES * ADI group 2 | 1.04 | (0.89, 1,21) | 1.05 | (0.92, 1.20) |
| Lower indiv. SES * ADI group 3 | 1.05 | (0.90, 1.24) | 1.04 | (0.92, 1.17) |
| Lower indiv. SES * ADI group 4 | 1.05 | (0.91, 1.21) | 0.99 | (0.88, 1.12) |
| Lower indiv. SES * ADI group 5 | 0.97 | (0.83, 1.12) | 1.02 | (0.91, 1.15) |
| Lower indiv. SES * ADI group 6 | 0.91 | (0.79, 1.06) | 0.95 | (0.85, 1.06) |
| Lower indiv. SES * ADI group 7 | 0.96 | (0.84, 1.10) | 0.96 | (0.83, 1.10) |
| Lower indiv. SES * ADI group 8 | 0.89 | (0.78, 1.03) | 0.96 | (0.85, 1.07) |
| Lower indiv. SES * ADI group 9 | 0.84** | (0.72, 0.98) | 0.86 ** | (0.76, 0.98) |
| Lower indiv. SES * ADI group 10 | 0.83** | (0.71, 0.96) | 0.79 *** | (0.69, 0.91) |
| Age [ref: 65-69] |  |  |  |  |
| 70-74 | 1.47*** | (1.42, 1.52) | 1.48 *** | (1.42, 1.54) |
| 75-79 | 2.15*** | (2.07, 2.24) | 2.19 *** | (2.10, 2.28) |
| 80-84 | 3.19*** | (3.04, 3.34) | 3.23 *** | (3.10, 3.37) |
| 85+ | 6.78*** | (6.41, 7.16) | 6.73 *** | (6.47, 7.01) |
| Sex [ref: male] |  |  |  |  |
| Female | 0.61*** | (0.59, 0.63) | 0.61 *** | (0.60, 0.63) |
| Race/Ethnicity [ref: white] |  |  |  |  |
| Black | 0.85*** | (0.80, 0.89) | 0.84 *** | (0.80, 0.88) |
| Hispanic | 0.66*** | (0.61, 0.72) | 0.64 *** | (0.61, 0.68) |
| Asian | 0.48*** | (0.41, 0.57) | 0.51 *** | (0.46, 0.55) |
| Other | 1.06* | (1.00, 1.13) | 1.05 | (0.98, 1.13) |
| # of chronic conditions [ref: none] |  |  |  |  |
| 1-2 | 1.07* | (1.00, 1.15) | 1.07 ** | (1.01, 1.15) |
| 3-5 | 1.34*** | (1.25, 1.43) | 1.33 *** | (1.25, 1.42) |
| 6+ | 2.25*** | (2.10, 2.43) | 2.22 *** | (2.08, 2.37) |
| BMI [ref: normal/overweight] |  |  |  |  |
| Obese | 0.77*** | (0.75, 0.80) | 0.76 *** | (0.74, 0.78) |
| Underweight | 2.30*** | (2.20, 2.40) | 2.31 *** | (2.22, 2.41) |
| Difficulties in ADL [ref: none] |  |  |  |  |
| 1+ | 2.84*** | (2.73, 2.95) | 2.80 *** | (2.72, 2.88) |
| Smoking status [ref: not smoking] |  |  |  |  |
| Smoke | 1.52*** | (1.45, 1.59) | 1.50 *** | (1.44, 1.57) |
| Survey year [ref: 2014] |  |  |  |  |
| 2015 | 0.97** | (0.95, 1.00) | 0.97 *** | (0.95, 0.99) |
| Constant | 0.02*** | (0.02, 0.02) | 0.02*** | (0.02, 0.02) |
| Observations | 468,741 | | 468,741 | |

Note: *p<0.1; **p<0.05; ***p<0.01. Source: Medicare Health Outcomes Survey 2014-2015.
