# Supplementary material for: Relationship of neighborhood and individual socioeconomic status on mortality among older adults: Evidence from cross-level interaction analyses
Source: PLoS One. 2022 May 19;17(5):e0267542. doi: 10.1371/journal.pone.0267542 (PMC9119539; doi:10.1371/journal.pone.0267542)
Supplement: S3 Table — Source: Medicare Health Outcomes Survey 2014–2015. (DOCX) [file pone.0267542.s004.docx]

**S3 Table. Descriptive characteristics comparing those with valid income values and those with missing income.**

|  |  | Income Valid | Income Missing |
| --- | --- | --- | --- |
|  | All | Model 1 Sample | Model 1 Excluded |
| n | 468741 | 328693 | 140048 |
| Died (%) | 35650 (7.6) | 22060 (6.7) | 13590 (9.7) |
| Homeownership (%) |  |  |  |
| Homeowner | 267925 (57.2) | 220118 (67.0) | 47807 (34.1) |
| Not owned | 129597 (27.6) | 97832 (29.8) | 31765 (22.7) |
| NA | 71219 (15.2) | 10743 (3.3) | 60476 (43.2) |
| ADI decile (%) |  |  |  |
| ADI group 1 | 37142 (7.9) | 25611 (7.8) | 11531 (8.2) |
| ADI group 2 | 44559 (9.5) | 31061 (9.4) | 13498 (9.6) |
| ADI group 3 | 53244 (11.4) | 37602 (11.4) | 15642 (11.2) |
| ADI group 4 | 55154 (11.8) | 39185 (11.9) | 15969 (11.4) |
| ADI group 5 | 55824 (11.9) | 39736 (12.1) | 16088 (11.5) |
| ADI group 6 | 53106 (11.3) | 37444 (11.4) | 15662 (11.2) |
| ADI group 7 | 48840 (10.4) | 34556 (10.5) | 14284 (10.2) |
| ADI group 8 | 43856 (9.4) | 30917 (9.4) | 12939 (9.2) |
| ADI group 9 | 39345 (8.4) | 27334 (8.3) | 12011 (8.6) |
| ADI group 10 | 37671 (8.0) | 25247 (7.7) | 12424 (8.9) |
| Age (%) |  |  |  |
| 65-69 | 139224 (29.7) | 103799 (31.6) | 35425 (25.3) |
| 70-74 | 125694 (26.8) | 90760 (27.6) | 34934 (24.9) |
| 75-79 | 89152 (19.0) | 61705 (18.8) | 27447 (19.6) |
| 80-84 | 61424 (13.1) | 40308 (12.3) | 21116 (15.1) |
| 85+ | 53247 (11.4) | 32121 (9.8) | 21126 (15.1) |
| Female (%) | 275528 (58.8) | 186263 (56.7) | 89265 (63.7) |
| Race/Ethnicity (%) |  |  |  |
| White | 296989 (63.4) | 240484 (73.2) | 56505 (40.3) |
| Asian | 15293 (3.3) | 12010 (3.7) | 3283 (2.3) |
| Black | 41986 (9.0) | 30348 (9.2) | 11638 (8.3) |
| Hispanic | 44520 (9.5) | 32326 (9.8) | 12194 (8.7) |
| Other | 12736 (2.7) | 9373 (2.9) | 3363 (2.4) |
| NA | 57217 (12.2) | 4152 (1.3) | 53065 (37.9) |
| # of chronic conditions (%) |  |  |  |
| None | 37526 (8.0) | 28412 (8.6) | 9114 (6.5) |
| 1 to 2 | 159908 (34.1) | 123932 (37.7) | 35976 (25.7) |
| 3 to 5 | 173125 (36.9) | 135009 (41.1) | 38116 (27.2) |
| 6 or more | 50537 (10.8) | 39447 (12.0) | 11090 (7.9) |
| NA | 47645 (10.2) | 1893 (0.6) | 45752 (32.7) |
| BMI (%) |  |  |  |
| Normal/Overweight | 254482 (54.3) | 201831 (61.4) | 52651 (37.6) |
| Obese | 121124 (25.8) | 96844 (29.5) | 24280 (17.3) |
| Underweight | 19309 (4.1) | 14542 (4.4) | 4767 (3.4) |
| NA | 73826 (15.7) | 15476 (4.7) | 58350 (41.7) |
| Difficulties in ADL (%) |  |  |  |
| None | 271544 (57.9) | 209724 (63.8) | 61820 (44.1) |
| 1+ | 152465 (32.5) | 116014 (35.3) | 36451 (26.0) |
| NA | 44732 (9.5) | 2955 (0.9) | 41777 (29.8) |
| Smoking status (%) |  |  |  |
| Do not smoke | 372591 (79.5) | 289633 (88.1) | 82958 (59.2) |
| Smoke | 40448 (8.6) | 32607 (9.9) | 7841 (5.6) |
| NA | 55702 (11.9) | 6453 (2.0) | 49249 (35.2) |
| Survey year 2015 (%) | 228547 (48.8) | 158516 (48.2) | 70031 (50.0) |

Source: Medicare Health Outcomes Survey 2014-2015.
