# Supplementary material for: Relationship of neighborhood and individual socioeconomic status on mortality among older adults: Evidence from cross-level interaction analyses
Source: PLoS One. 2022 May 19;17(5):e0267542. doi: 10.1371/journal.pone.0267542 (PMC9119539; doi:10.1371/journal.pone.0267542)
Supplement: S4 Table — Source: Medicare Health Outcomes Survey 2014–2015. (DOCX) [file pone.0267542.s005.docx]

**S4 Table. Descriptive characteristics comparing those with valid homeownership values and those with missing homeownership.**

|  |  |  |  |
| --- | --- | --- | --- |
|  | Overall | Homeownership Valid | Homeownership Missing |
| n | 468741 | 397522 (84.8) | 71219 (15.2) |
| Died (%) | 35650 (7.6) | 26448 (6.7) | 9202 (12.9) |
| Income (%) |  |  |  |
| Higher-income | 207586 (44.3) | 203565 (51.2) | 4021 (5.6) |
| Low-income | 121107 (25.8) | 114385 (28.8) | 6722 (9.4) |
| NA | 140048 (29.9) | 79572 (20.0) | 60476 (84.9) |
| ADI decile (%) |  |  |  |
| ADI group 1 | 37142 (7.9) | 31330 (7.9) | 5812 (8.2) |
| ADI group 2 | 37671 (8.0) | 37876 (9.5) | 6683 (9.4) |
| ADI group 3 | 44559 (9.5) | 45509 (11.4) | 7735 (10.9) |
| ADI group 4 | 53244 (11.4) | 47099 (11.8) | 8055 (11.3) |
| ADI group 5 | 55154 (11.8) | 47622 (12.0) | 8202 (11.5) |
| ADI group 6 | 55824 (11.9) | 45207 (11.4) | 7899 (11.1) |
| ADI group 7 | 53106 (11.3) | 41440 (10.4) | 7400 (10.4) |
| ADI group 8 | 48840 (10.4) | 37180 (9.4) | 6676 (9.4) |
| ADI group 9 | 43856 (9.4) | 33268 (8.4) | 6077 (8.5) |
| ADI group 10 | 39345 (8.4) | 30991 (7.8) | 6680 (9.4) |
| Age (%) |  |  |  |
| 65-69 | 139224 (29.7) | 121764 (30.6) | 17460 (24.5) |
| 70-74 | 125694 (26.8) | 108992 (27.4) | 16702 (23.5) |
| 75-79 | 89152 (19.0) | 75775 (19.1) | 13377 (18.8) |
| 80-84 | 61424 (13.1) | 50650 (12.7) | 10774 (15.1) |
| 85+ | 53247 (11.4) | 40341 (10.1) | 12906 (18.1) |
| Female (%) | 275528 (58.8) | 232474 (58.5) | 43054 (60.5) |
| Race/Ethnicity (%) |  |  |  |
| White | 296989 (63.4) | 284719 (71.6) | 12270 (17.2) |
| Asian | 18007 (3.8) | 14632 (3.7) | 661 (0.9) |
| Black | 41986 (9.0) | 39326 (9.9) | 2660 (3.7) |
| Hispanic | 12643 (2.7) | 41521 (10.4) | 2999 (4.2) |
| Other | 12652 (2.7) | 11886 (3.0) | 850 (1.2) |
| NA | 86464 (18.4) | 5438 (1.4) | 51779 (72.7) |
| # of chronic conditions (%) |  |  |  |
| None | 37526 (8.0) | 33261 (8.4) | 4265 (6.0) |
| 1 to 2 | 159908 (34.1) | 147667 (37.1) | 12241 (17.2) |
| 3 to 5 | 173125 (36.9) | 165901 (41.7) | 7224 (10.1) |
| 6 or more | 50537 (10.8) | 48337 (12.2) | 2200 (3.1) |
| NA | 47645 (10.2) | 2356 (0.6) | 45289 (63.6) |
| BMI (%) |  |  |  |
| Normal/Overweight | 254482 (54.3) | 242691 (61.1) | 11791 (16.6) |
| Obese | 121124 (25.8) | 115446 (29.0) | 5678 (8.0) |
| Underweight | 19309 (4.1) | 17962 (4.5) | 1347 (1.9) |
| NA | 73826 (15.7) | 21423 (5.4) | 52403 (73.6) |
| Difficulties in ADL (%) |  |  |  |
| None | 271544 (57.9) | 255664 (64.3) | 15880 (22.3) |
| 1+ | 152465 (32.5) | 138424 (34.8) | 14041 (19.7) |
| NA | 44732 (9.5) | 3434 (0.9) | 41298 (58.0) |
| Smoking status (%) |  |  |  |
| Do not smoke | 372591 (79.5) | 351305 (88.4) | 21286 (29.9) |
| Smoke | 40448 (8.6) | 38323 (9.6) | 2125 (3.0) |
| NA | 55702 (11.9) | 7894 (2.0) | 47808 (67.1) |
| Survey year 2015 (%) | 228547 (48.8) | 192051 (48.3) | 36496 (51.2) |

Source: Medicare Health Outcomes Survey 2014-2015.
