# Supplementary material for: Relationship of neighborhood and individual socioeconomic status on mortality among older adults: Evidence from cross-level interaction analyses
Source: PLoS One. 2022 May 19;17(5):e0267542. doi: 10.1371/journal.pone.0267542 (PMC9119539; doi:10.1371/journal.pone.0267542)
Supplement: S5 Table — Source: Medicare Health Outcomes Survey 2014–2015. (DOCX) [file pone.0267542.s006.docx]

**S5 Table. Regression results with and without interaction terms between income/homeownership and neighborhood status**.

|  | Model 1: Income | | | | Model 2: Homeownership | | | |
| --- | --- | --- | --- | --- | --- | --- | --- | --- |
|  | Regression 1-1 | | Regression 1-2 | | Regression 2-1 | | Regression 2-2 | |
| Variables | Odds Ratio | 95% Confidence Interval | Odds Ratio | 95% Confidence Interval | Odds Ratio | 95% Confidence Interval | Odds Ratio | 95% Confidence Interval |
| Income level [ref: higher-income] |  |  |  |  |  |  |  |  |
| Low-income | 1.28*** | (1.21 - 1.36) | 1.40*** | (1.19 - 1.65) | - | - | - | - |
| Homeownership [ref: homeowner] |  |  |  |  |  |  |  |  |
| Nonhomeowner | - | - | - | - | 1.40*** | (1.35 - 1.47) | 1.47*** | (1.32 - 1.63) |
| ADI decile [ref: group 1 (least disadvantaged)] |  |  |  |  |  |  |  |  |
| ADI group 2 | 0.97 | (0.91 - 1.04) | 0.95 | (0.87 - 1.04) | 0.96 | (0.91 - 1.03) | 0.93 | (0.84 - 1.03) |
| ADI group 3 | 0.98 | (0.91 - 1.06) | 0.97 | (0.87 - 1.07) | 1.01 | (0.93 - 1.08) | 0.99 | (0.89 - 1.10) |
| ADI group 4 | 1.07* | (0.99 - 1.15) | 1.05 | (0.96 - 1.15) | 1.09** | (1.02 - 1.16) | 1.08* | (0.99 - 1.19) |
| ADI group 5 | 1.11** | (1.02 - 1.20) | 1.14*** | (1.04 - 1.25) | 1.13*** | (1.05 - 1.22) | 1.12** | (1.01 - 1.25) |
| ADI group 6 | 1.09* | (1.00 - 1.19) | 1.14*** | (1.04 - 1.24) | 1.13*** | (1.05 - 1.22) | 1.15*** | (1.04 - 1.28) |
| ADI group 7 | 1.10** | (1.01 - 1.19) | 1.13** | (1.03 - 1.25) | 1.14*** | (1.06 - 1.22) | 1.17*** | (1.07 - 1.27) |
| ADI group 8 | 1.16*** | (1.05 - 1.29) | 1.28*** | (1.13 - 1.44) | 1.25*** | (1.14 - 1.36) | 1.28*** | (1.14 - 1.43) |
| ADI group 9 | 1.11** | (1.01 - 1.21) | 1.29*** | (1.17 - 1.41) | 1.23*** | (1.14 - 1.32) | 1.33*** | (1.21 - 1.47) |
| ADI group 10 (most disadvantaged) | 1.10** | (1.02 - 1.19) | 1.29*** | (1.16 - 1.44) | 1.17*** | (1.09 - 1.26) | 1.38*** | (1.20 - 1.58) |
| Interaction |  |  |  |  |  |  |  |  |
| Low-income * ADI group 2 |  |  | 1.06 | (0.95 - 1.19) |  |  | 1.09 | (0.95 - 1.25) |
| Low-income * ADI group 3 |  |  | 1.04 | (0.88 - 1.24) |  |  | 1.05 | (0.93 - 1.18) |
| Low-income * ADI group 4 |  |  | 1.04 | (0.88 - 1.22) |  |  | 1.02 | (0.92 - 1.13) |
| Low-income * ADI group 5 |  |  | 0.92 | (0.77 - 1.11) |  |  | 1.02 | (0.90 - 1.14) |
| Low-income * ADI group 6 |  |  | 0.89 | (0.73 - 1.09) |  |  | 0.96 | (0.83 - 1.12) |
| Low-income * ADI group 7 |  |  | 0.91 | (0.76 - 1.09) |  |  | 0.94 | (0.85 - 1.05) |
| Low-income * ADI group 8 |  |  | 0.82** | (0.69 - 0.98) |  |  | 0.94 | (0.82 - 1.07) |
| Low-income * ADI group 9 |  |  | 0.75*** | (0.62 - 0.92) |  |  | 0.83*** | (0.74 - 0.93) |
| Low-income * ADI group 10 |  |  | 0.77*** | (0.65 - 0.90) |  |  | 0.76*** | (0.62 - 0.92) |
| Age [ref: 65-69] |  |  |  |  |  |  |  |  |
| 70-74 | 1.47*** | (1.41 - 1.53) | 1.47*** | (1.41 - 1.53) | 1.50*** | (1.43 - 1.56) | 1.49*** | (1.43 - 1.56) |
| 75-79 | 2.15*** | (2.05 - 2.25) | 2.15*** | (2.05 - 2.26) | 2.17*** | (2.08 - 2.27) | 2.16*** | (2.07 - 2.26) |
| 80-84 | 3.14*** | (2.96 - 3.33) | 3.14*** | (2.95 - 3.33) | 3.20*** | (3.04 - 3.37) | 3.19*** | (3.03 - 3.35) |
| 85+ | 6.57*** | (6.13 - 7.05) | 6.55*** | (6.11 - 7.03) | 6.36*** | (6.05 - 6.70) | 6.31*** | (6.00 - 6.65) |
| Sex [ref: male] |  |  |  |  |  |  |  |  |
| Female | 0.60*** | (0.58 - 0.63) | 0.60*** | (0.58 - 0.63) | 0.60*** | (0.58 - 0.61) | 0.59*** | (0.58 - 0.61) |
| Race/Ethnicity [ref: white] |  |  |  |  |  |  |  |  |
| Black | 0.85*** | (0.80 - 0.90) | 0.85*** | (0.80 - 0.90) | 0.85*** | (0.81 - 0.90) | 0.85*** | (0.81 - 0.89) |
| Hispanic | 0.67*** | (0.60 - 0.75) | 0.67*** | (0.60 - 0.75) | 0.68*** | (0.61 - 0.76) | 0.68*** | (0.61 - 0.75) |
| Asian | 0.49*** | (0.39 - 0.61) | 0.48*** | (0.38 - 0.60) | 0.50*** | (0.41 - 0.61) | 0.50*** | (0.41 - 0.61) |
| Other | 1.03 | (0.93 - 1.16) | 1.03 | (0.93 - 1.15) | 1.07* | (0.99 - 1.16) | 1.07 | (0.99 - 1.16) |
| # of chronic conditions [ref: none] |  |  |  |  |  |  |  |  |
| 1-2 | 1.17*** | (1.08 - 1.26) | 1.17*** | (1.08 - 1.26) | 1.16*** | (1.08 - 1.25) | 1.16*** | (1.08 - 1.25) |
| 3-5 | 1.51*** | (1.40 - 1.62) | 1.51*** | (1.40 - 1.62) | 1.50*** | (1.40 - 1.62) | 1.50*** | (1.39 - 1.62) |
| 6+ | 2.59*** | (2.41 - 2.79) | 2.58*** | (2.40 - 2.78) | 2.54*** | (2.35 - 2.74) | 2.53*** | (2.35 - 2.73) |
| BMI [ref: normal/overweight] |  |  |  |  |  |  |  |  |
| Obese | 0.78*** | (0.75 - 0.81) | 0.78*** | (0.75 - 0.80) | 0.77*** | (0.75 - 0.80) | 0.77*** | (0.75 - 0.80) |
| Underweight | 2.37*** | (2.25 - 2.49) | 2.37*** | (2.25 - 2.49) | 2.29*** | (2.19 - 2.40) | 2.29*** | (2.19 - 2.40) |
| Difficulties in ADL [ref: none] |  |  |  |  |  |  |  |  |
| 1+ | 2.89*** | (2.78 - 3.00) | 2.88*** | (2.77 - 2.99) | 2.71*** | (2.63 - 2.78) | 2.70*** | (2.62 - 2.78) |
| Smoking status [ref: not smoking] |  |  |  |  |  |  |  |  |
| Smoke | 1.57*** | (1.49 - 1.65) | 1.56*** | (1.49 - 1.65) | 1.59*** | (1.53 - 1.66) | 1.59*** | (1.53 - 1.66) |
| Survey year [ref: 2014] |  |  |  |  |  |  |  |  |
| 2015 | 0.96*** | (0.93 - 0.98) | 0.96*** | (0.93 - 0.98) | 0.97** | (0.94 - 1.00) | 0.97** | (0.94 - 1.00) |
| Constant | 0.01*** | (0.01 - 0.02) | 0.01*** | (0.01 - 0.02) | 0.01*** | (0.01 - 0.02) | 0.01*** | (0.01 - 0.02) |
| Observations | 302,566 | | 302,566 | | 362,609 | | 362,609 | |

Source: Medicare Health Outcomes Survey 2014-2015.
