# Supplementary material for: Relationship of neighborhood and individual socioeconomic status on mortality among older adults: Evidence from cross-level interaction analyses
Source: PLoS One. 2022 May 19;17(5):e0267542. doi: 10.1371/journal.pone.0267542 (PMC9119539; doi:10.1371/journal.pone.0267542)
Supplement: S6 Table — Source: Medicare Health Outcomes Survey 2014–2015. (DOCX) [file pone.0267542.s007.docx]

| Annual Income | Average Mortality (%) | N |
| --- | --- | --- |
| <$10,000 | 9.3 | 50,608 |
| $10,000-$20,000 | 8.7 | 70,499 |
| $20,000-$30,000 | 7.4 | 57,264 |
| $30,000-$50,000 | 5.6 | 72,829 |
| >$50,000 | 3.7 | 77,493 |
| Total |  | 328,693 |
